# Crystal structure and Hirshfeld surface analysis of 1-[(1-butyl-1*H*-1,2,3-triazol-4-yl)meth­yl]-3-methyl­quinoxalin-2(1*H*)-one

**DOI:** 10.1107/S205698901801589X

**Published:** 2018-11-20

**Authors:** Nadeem Abad, Youssef Ramli, Tuncer Hökelek, Nada Kheira Sebbar, Joel T. Mague, El Mokhtar Essassi

**Affiliations:** a Laboratoire de Chimie Organique Hétérocyclique URAC 21, Pôle de Compétence Pharmacochimie, Av. Ibn Battouta, BP 1014, Faculté des Sciences, Université Mohammed V, Rabat, Morocco; b Laboratory of Medicinal Chemistry, Faculty of Medicine and Pharmacy, Mohammed V University, Rabat, Morocco; c Department of Physics, Hacettepe University, 06800 Beytepe, Ankara, Turkey; d Laboratoire de Chimie Bioorganique Appliquée, Faculté des Sciences, Université Ibn Zohr, Agadir, Morocco; e Department of Chemistry, Tulane University, New Orleans, LA 70118, USA

**Keywords:** crystal structure, di­hydro­quinoxaline, triazole, π-stacking, Hirshfeld surface

## Abstract

The title compound, C_16_H_19_N_5_O, is built up from a planar quinoxalinone ring system linked through a methyl­ene bridge to a 1,2,3-triazole ring, which in turn carries an *n*-butyl substituent. The triazole ring is inclined by 67.09 (4)° to the quinoxalinone ring plane. In the crystal, the mol­ecules form oblique stacks along the *a*-axis direction through inter­molecular C—H_Trz_⋯N_Trz_ (Trz = triazole) hydrogen bonds, and offset π-stacking inter­actions between quinoxalinone rings [centroid–centroid distance = 3.9107 (9) Å] and π–π inter­actions, which are associated pairwise by inversion-related C—H_Dhydqn_⋯π(ring) (Dhydqn = di­hydro­quinoxaline) inter­actions. The Hirshfeld surface analysis of the crystal structure indicates that the most important contributions for the crystal packing are from H⋯H (52.7%), H⋯N/N⋯H (18.9%) and H⋯C/C⋯H (17.0%) inter­actions.

## Abstract 2

The title compound is built up from a planar quinoxalinone ring system linked through a methyl­ene bridge to a 1,2,3-triazole ring, which is inclined by 67.09 (4)° to the quinoxalinone ring plane.

## Chemical context   

Quinoxaline groups are well known, important nitrogen-containing heterocyclic compounds comprising a benzene and a pyrazine ring fused together. Diversely substituted quinoxalines and their derivatives embedded with variety of functional groups are important biological agents and a significant amount of research activity has been directed towards this class of compounds. These mol­ecules exhibit a wide range of biological applications and are potentially useful in medicinal chemistry research and have therapeutic applications such as anti­microbial (Attia *et al.*, 2013 ▸; Vieira *et al.*, 2014 ▸; Teja *et al.*, 2016 ▸), anti-inflammatory (Guirado *et al.*, 2012 ▸), anti­cancer (Abbas *et al.*, 2015 ▸), anti­diabetic (Kulkarni *et al.*, 2012 ▸) and anti­histaminic activities (Sridevi *et al.*, 2010 ▸). As a continuation of our research works on the synthesis, spectroscopic and biological properties of quinoxaline derivatives (Ramli *et al.*, 2013 ▸, 2017 ▸; Ramli & Essassi, 2015 ▸; Abad *et al.*, 2018*a* ▸,*b* ▸,*c* ▸; Ellouz *et al.*, 2015 ▸; Sebbar *et al.*, 2014 ▸), we report herein the mol­ecular and crystal structures along with the Hirshfeld surface analysis of the title compound, 1-[(1-butyl-1*H*-1,2,3-triazol-5-yl)meth­yl]-3-methyl-1,2-di­hydro­quinoxalin-2-one.

## Structural commentary   

**Figure 1:**
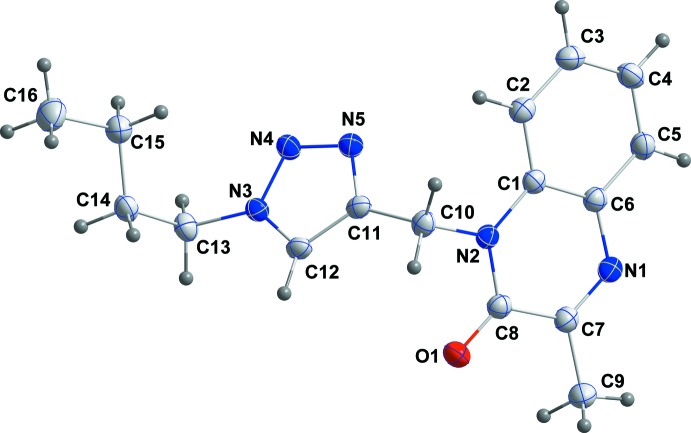
The title mol­ecule with labelling scheme and 50% probability ellipsoids.

**Figure 2:**
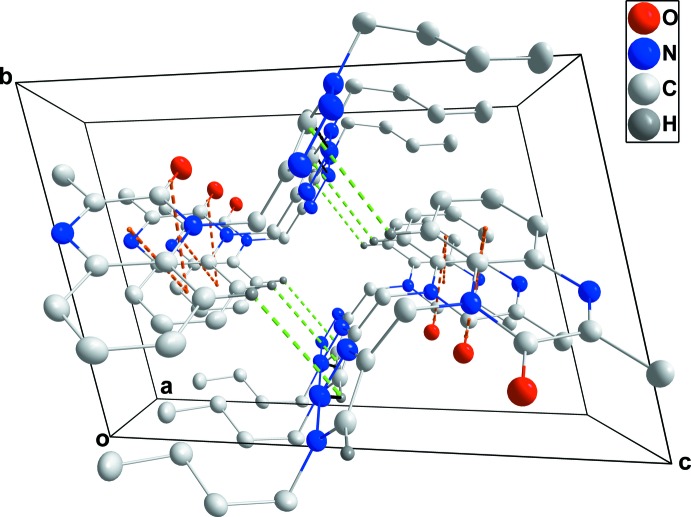
Packing viewed along the *a*-axis direction. C—H⋯N hydrogen bonds are shown by black dashed lines while π-stacking and C—H⋯π(ring) inter­actions are shown, respectively, by orange and green dashed lines.

The title compound, (I), is built up from the two fused six-membered rings of a quinoxalinone moiety linked through a methyl­ene bridge to a 1,2,3-triazole ring, which in turn carries an *n*-butyl substituent on N3 (Fig. 1 ▸). The di­hydro­quinoxaline unit is planar within 0.029 (1) Å (r.m.s. deviation of the fitted atoms = 0.0123 Å) and the triazole ring is inclined by 67.09 (4)° to the above-mentioned plane. The mol­ecule adopts a Z-shaped conformation with the (1*H*-1,2,3-triazol-5-yl)methyl substituent projecting well out of the mean plane of the di­hydro­quinxalone unit, as indicated by the C1—N2—C10—C11 torsion angle of 90.85 (16)°. The *n*-butyl group is oriented in the opposite direction as seen from the N4—N3—C13—C14 torsion angle of −95.26 (16)° (Fig. 2 ▸).
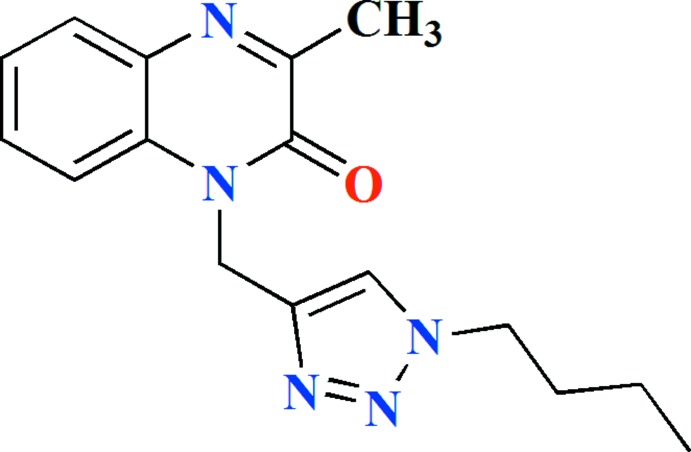


## Supra­molecular features   

Hydrogen bonding and van der Waals contacts are the dominant inter­actions in the crystal packing. In the crystal, the mol­ecules form oblique stacks along the *a*-axis direction through inter­molecular C—H_Trz_⋯N_Trz_ (Trz = triazole) hydrogen bonds (Table 1 ▸), and offset, very weak π-stacking inter­actions between the *A* (C1–C6) and *B* (N1/N2/C1/C6–C8) rings [centroid–centroid distance = 3.9107 (9) Å, dihedral angle = 0.94 (7)°] and π-inter­actions between the C8=O1 carbonyl group and the *B* rings [O1—centroid = 3.5505 (14) Å, C8–centroid = 3.4546 (17) Å, C8=O1⋯centroid = 75.51 (9)°]. Pairs of stacks are associated through C—H_Dhydqn_⋯π (Dhydqn = di­hydro­quinoxaline) inter­actions, generating small, diamond-shaped channels along the *a*-axis direction (Table 1 ▸ and Fig. 2 ▸).

## Database Survey   

A search of the CSD (Version 5.39, updated May 2018; Groom *et al.*, 2016 ▸) using the fragment shown in Scheme 2 (*R* = C, *R*1 = nothing) generated 37 hits. Of these, the ones most comparable to the title mol­ecule have *R*1 = CH_3_ and *R* = CH_2_C≡CH (Benzeid *et al.*, 2009 ▸), CH_2_Ph (Ramli *et al.*, 2010*a* ▸, 2018 ▸), C_2_H_5_ (Benzeid *et al.*, 2008 ▸), (1,3-oxazolidin-3-yl)ethyl (Caleb *et al.*, 2009 ▸), CH_2_CH=CH_2_ (Ramli *et al.*, 2010*b* ▸) and the isomer with *R* = (1-butyl-1*H*-1,2,3- triazol-5-yl)methyl (Abad *et al.*, 2018*a* ▸). Those with *R* = CH_2_C≡CH and C_2_H_5_ have *Z*′ = 1. A common feature of the above subset as well as the majority of the other compounds with different *R*1 substituents is the geometry of the bicyclic unit, which is either planar or has a slight end-to-end twist. Another feature is the orientation of the *R* group, which generally has a C—N—C—C torsion angle >65° and in quite a few cases, this is close to 90°. A comparison of the conformation of the title mol­ecule with that of its (1-butyl-1*H*-1,2,3-triazol-5-yl)methyl isomer shows that the latter has a U shape with the *R* group extending back over the bicyclic unit as the result of an intra­molecular C—H⋯O hydrogen bond from the α hydrogen of the butyl group while in the former, the more remote position of the butyl group on the triazole ring disfavours such an inter­action and the mol­ecule adopts a Z shape. This conformation is favoured by the opportunity for π-stacking and C—H⋯π(ring) inter­actions in the crystal.
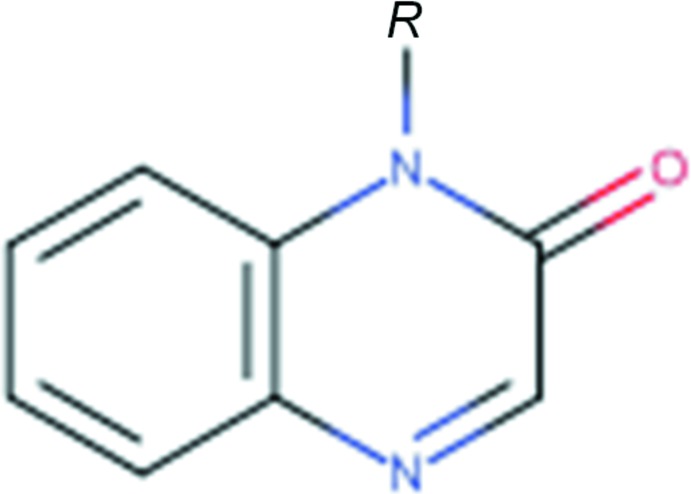


## Hirshfeld surface analysis   

**Figure 3:**
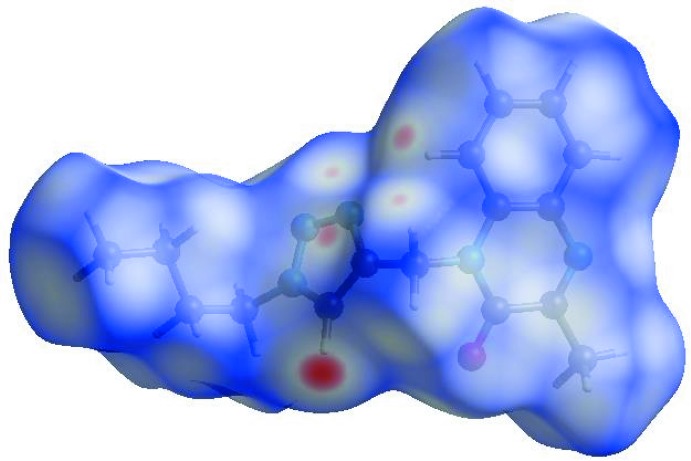
View of the three-dimensional Hirshfeld surface of the title compound plotted over *d*_norm_ in the range −0.2380 to 1.1723 a.u.

**Figure 4:**
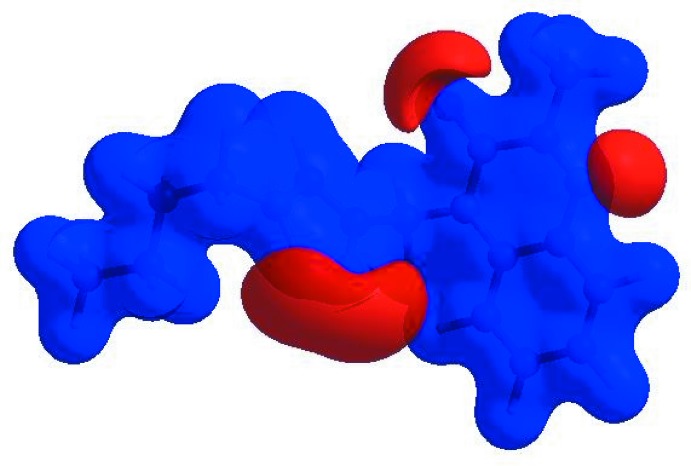
View of the three-dimensional Hirshfeld surface of the title compound plotted over electrostatic potential energy in the range −0.0500 to 0.0500 a.u. using the STO-3 G basis set at the Hartree–Fock level of theory. Hydrogen-bond donors and acceptors are shown as blue and red regions around the atoms corresponding to positive and negative potentials, respectively.

**Figure 5:**
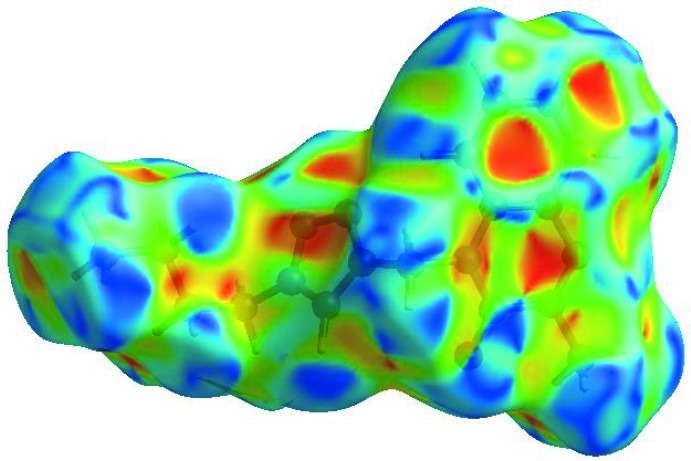
Hirshfeld surface of the title compound plotted over shape-index.

**Figure 6:**
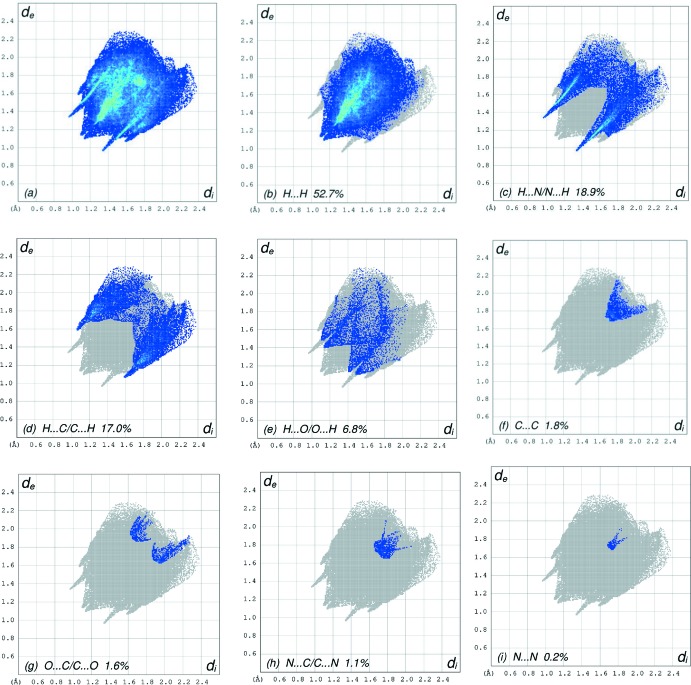
The full two-dimensional fingerprint plots for the title compound, showing (*a*) all inter­actions, and delineated into (*b*) H⋯H, (*c*) H⋯N/N⋯H, (*d*) H⋯C/C⋯H, (*e*) H⋯O/O⋯H, (*f*) C⋯C, (*g*) O⋯C/C⋯O, (*h*) N⋯C/C⋯N and (*i*) N⋯N inter­actions. The *d*_i_ and *d*_e_ values are the closest inter­nal and external distances (in Å) from given points on the Hirshfeld surface contacts.

**Figure 7:**
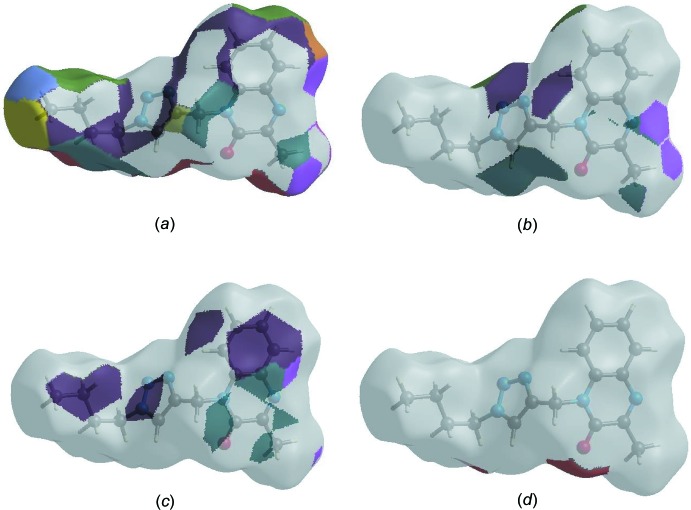
The Hirshfeld surface representations with the function *d*_norm_ plotted onto the surface for (*a*) H⋯H, (*b*) H⋯N/N⋯H, (*c*) H⋯C/C⋯H and (*d*) H⋯O/O⋯H inter­actions.

In order to visualize the inter­molecular inter­actions in the crystal of the title compound, a Hirshfeld surface (HS) analysis (Hirshfeld, 1977 ▸; Spackman & Jayatilaka, 2009 ▸) was carried out using *CrystalExplorer17.5* (Turner *et al.*, 2017 ▸). In the HS plotted over *d*_norm_ (Fig. 3 ▸), the white surface indicates contacts with distances equal to the sum of van der Waals radii, and the red and blue colours indicate distances shorter (in close contact) or longer (distant contact) than the sum of the van der Waals radii, respectively (Venkatesan *et al.*, 2016 ▸). The bright-red spots appearing near the hydrogen atom H12 indicates their role as the respective donors and/or acceptors in the dominant C—H⋯N hydrogen bonds; they also appear as blue and red regions corresponding to positive and negative potentials on the HS mapped over electrostatic potential (Spackman *et al.*, 2008 ▸; Jayatilaka *et al.*, 2005 ▸) as shown in Fig. 4 ▸. The blue regions indicate positive electrostatic potential (hydrogen-bond donors), while the red regions indicate negative electrostatic potential (hydrogen-bond acceptors). The shape-index of the HS is a tool to visualize the π–π stacking by the presence of adjacent red and blue triangles; if there are no adjacent red and/or blue triangles, then there are no π–π inter­actions. Fig. 5 ▸ clearly suggest that there are π–π inter­actions in (I). The overall two-dimensional fingerprint plot, Fig. 6 ▸(*a*), and those delineated into H⋯H, H⋯N/N⋯H, H⋯C/C⋯H, H⋯O/O⋯H, C⋯C, O⋯C/C⋯O, N⋯C/C⋯N and N⋯N contacts (McKinnon *et al.*, 2007 ▸) are illus­trated in Fig. 6 ▸(*b*)–(*i*), respectively, together with their relative contributions to the Hirshfeld surface. The most important inter­action is H⋯H contributing 52.7% to the overall crystal packing, which is reflected in Fig. 6 ▸(*b*) as widely scattered points of high density due to the large hydrogen content of the mol­ecule. The split spike with the tip at *d*_e_ = *d*_i_ = 1.13 Å in Fig. 6 ▸(*b*) is due to the short inter­atomic H⋯H contacts (Table 2 ▸). The pair of characteristic wings resulting in the fingerprint plot delineated into H⋯N/N⋯H contacts Fig. 6 ▸(*c*), contribute 18.9% to the HS (Table 2 ▸) and are viewed as pair of spikes with the tips at *d*_e_ + *d*_i_ = 2.23 Å. In the presence of weak C—H⋯π inter­actions (Table 1 ▸) in the crystal, the pair of characteristic wings resulting in the fingerprint plot delineated into H⋯C/C⋯H contacts with a 17.0% contribution to the HS have a symmetrical distribution of points, Fig. 7 ▸(*d*), with the tips at *d*_e_ + *d*_i_ = 2.65 Å (Table 2 ▸). Finally, the H⋯O/O⋯H [Fig. 6 ▸(*e*)] contacts (Table 2 ▸) in the structure with 6.8% contribution to the HS also have symmetrical distribution of points, namely two pairs of thin and thick edges at *d*_e_ + *d*_i_ ∼2.53 and 2.58 Å, respectively.

The Hirshfeld surface representations with the function *d*_norm_ plotted onto the surface are shown for the H⋯H, H⋯N/N⋯H, H⋯C/C⋯H and H⋯O/O⋯H inter­actions in Fig. 7 ▸(*a*)–(*d*), respectively.

The Hirshfeld surface analysis confirms the importance of H-atom contacts in establishing the packing. The large number of H⋯H, H⋯N/N⋯H, H⋯C/C⋯H and H⋯O/O⋯H inter­actions suggests that van der Waals inter­actions and hydrogen bonding play the major roles in the crystal packing (Hathwar *et al.*, 2015 ▸).

## Synthesis and crystallization   

To a solution of 3-methyl-1-(prop-2-yn­yl)-3,4-di­hydro­quinoxalin-2(1*H*)-one (0.68 mmol) in ethanol (15 mL) was added 1-azido­butane (1.03 mmol). The reaction mixture was stirred under reflux for 72 h. After completion of the reaction (monitored by TLC), the solution was concentrated and the residue was purified by column chromatography on silica gel by using as eluent the mixture (hexa­ne/ethyl acetate 8:2). The solid product obtained was crystallized from ethanol to afford colourless crystals in 78% yield.

## Refinement   

Crystal data, data collection and structure refinement details are summarized in Table 3 ▸. H atoms were located in a difference Fourier map and were freely refined.

## Supplementary Material

Crystal structure: contains datablock(s) I, global. DOI: 10.1107/S205698901801589X/xu5949sup1.cif

Structure factors: contains datablock(s) I. DOI: 10.1107/S205698901801589X/xu5949Isup2.hkl

Supporting information file. DOI: 10.1107/S205698901801589X/xu5949Isup3.cdx

Supporting information file. DOI: 10.1107/S205698901801589X/xu5949Isup4.cml

CCDC reference: 1878133

Additional supporting information:  crystallographic information; 3D view; checkCIF report

## Figures and Tables

**Table 1:** Hydrogen-bond geometry (Å, °) *Cg*1 is the centroid of the N3–N5/C11/C12 ring.

*D*—H⋯*A*	*D*—H	H⋯*A*	*D*⋯*A*	*D*—H⋯*A*
C12—H12⋯N4^iii^	0.954 (19)	2.419 (19)	3.2282 (19)	142.4 (15)
C2—H2⋯*Cg*1^vii^	0.966 (18)	2.986 (19)	3.642 (1)	126.3 (14)

Symmetry codes: (iii) 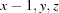; (vii) 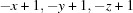.

**Table 2:** Selected interatomic distances (Å)

O1⋯C11	3.3134 (19)	C2⋯C10^iv^	3.586 (2)
O1⋯C13^i^	3.306 (2)	C2⋯C11	3.559 (2)
O1⋯C14^i^	3.193 (2)	C2⋯C11^vii^	3.589 (2)
O1⋯H10*B*	2.341 (17)	C3⋯C10^iv^	3.431 (2)
O1⋯H9*B*	2.75 (2)	C4⋯C8^iv^	3.502 (2)
O1⋯H9*C*	2.79 (2)	C5⋯C8^iv^	3.491 (2)
O1⋯H13*A* ^ii^	2.696 (17)	C5⋯C7^iv^	3.429 (2)
O1⋯H13*B* ^i^	2.62 (2)	C11⋯C13^ii^	3.589 (2)
O1⋯H14*A* ^i^	2.752 (18)	C12⋯C13^ii^	3.470 (2)
N1⋯N2	2.8013 (17)	C16⋯C16^viii^	3.577 (3)
N2⋯C3^iii^	3.421 (2)	C2⋯H15*B* ^vii^	2.991 (19)
N2⋯N5	3.1514 (17)	C2⋯H10*A*	2.600 (17)
N4⋯C15	3.364 (2)	C2⋯H10*B* ^iv^	2.928 (17)
N4⋯C12^iv^	3.228 (2)	C8⋯H13*A* ^ii^	2.918 (18)
N5⋯C2	3.343 (2)	C10⋯H2	2.608 (18)
N1⋯H5^v^	2.680 (18)	C11⋯H2^vii^	2.755 (19)
N3⋯H15*B*	2.860 (19)	C12⋯H13*A* ^ii^	2.973 (17)
N4⋯H12^iv^	2.42 (2)	H2⋯H10*A*	2.07 (2)
N4⋯H13*B* ^ii^	2.944 (19)	H4⋯H16*B* ^vi^	2.52 (3)
N4⋯H15*B*	2.936 (18)	H9*B*⋯H15*A* ^ii^	2.40 (3)
N4⋯H3^vi^	2.821 (18)	H13*A*⋯H15*A*	2.54 (3)
N5⋯H2	2.819 (19)	H14*A*⋯H16*A*	2.46 (3)
N5⋯H10*B* ^iv^	2.733 (17)	H14*B*⋯H16*C*	2.57 (3)
N5⋯H2^vii^	2.932 (18)	H15*A*⋯H13*A*	2.54 (3)
N5⋯H10*A* ^vii^	2.676 (18)	H15*A*⋯H9*B* ^ii^	2.40 (3)
N5⋯H13*B* ^ii^	2.93 (2)		

Symmetry codes: (i) 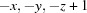; (ii) 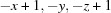; (iii) 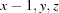; (iv) 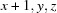; (v) 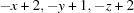; (vi) 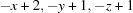; (vii) 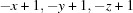; (viii) 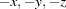.

**Table 3:** Experimental details

Crystal data	
Chemical formula	C_16_H_19_N_5_O
*M*_r_	297.36
Crystal system, space group	Triclinic, *P* 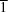
Temperature (K)	150
*a*, *b*, *c* (Å)	5.3265 (2), 9.9946 (4), 14.5414 (5)
α, β, γ (°)	103.054 (2), 100.039 (2), 93.108 (2)
*V* (Å^3^)	739.03 (5)
*Z*	2
Radiation type	Cu *K*α
μ (mm^−1^)	0.71
Crystal size (mm)	0.25 × 0.21 × 0.02

Data collection	
Diffractometer	Bruker D8 VENTURE PHOTON 100 CMOS
Absorption correction	Multi-scan (*SADABS*; Krause *et al.*, 2015 ▸)
*T*_min_, *T*_max_	0.84, 0.97
No. of measured, independent and observed [*I* > 2σ(*I*)] reflections	5735, 2764, 2281
*R*_int_	0.030
(sin θ/λ)_max_ (Å^−1^)	0.617

Refinement	
*R*[*F* ^2^ > 2σ(*F* ^2^)], *wR*(*F* ^2^), *S*	0.041, 0.100, 1.07
No. of reflections	2764
No. of parameters	276
H-atom treatment	All H-atom parameters refined
Δρ_max_, Δρ_min_ (e Å^−3^)	0.25, −0.21

Computer programs: *APEX3* and *SAINT* (Bruker, 2016 ▸), *SHELXT* (Sheldrick, 2015*a* ▸), *SHELXL2018* (Sheldrick, 2015*b* ▸), *DIAMOND* (Brandenburg & Putz, 2012 ▸) and *SHELXTL* (Sheldrick, 2008 ▸).

## supplementary crystallographic information

### Crystal data

**Table:**

C_16_H_19_N_5_O	*Z* = 2
*M**_r_* = 297.36	*F*(000) = 316
Triclinic, *P*1	*D*_x_ = 1.336 Mg m^−^^3^
*a* = 5.3265 (2) Å	Cu *K*α radiation, λ = 1.54178 Å
*b* = 9.9946 (4) Å	Cell parameters from 3995 reflections
*c* = 14.5414 (5) Å	θ = 3.2–72.1°
α = 103.054 (2)°	µ = 0.71 mm^−^^1^
β = 100.039 (2)°	*T* = 150 K
γ = 93.108 (2)°	Plate, colourless
*V* = 739.03 (5) Å^3^	0.25 × 0.21 × 0.02 mm

### Data collection

**Table:**

Bruker D8 VENTURE PHOTON 100 CMOS diffractometer	2764 independent reflections
Radiation source: INCOATEC IµS micro-focus source	2281 reflections with *I* > 2σ(*I*)
Mirror monochromator	*R*_int_ = 0.030
Detector resolution: 10.4167 pixels mm^-1^	θ_max_ = 72.1°, θ_min_ = 3.2°
ω scans	*h* = −6→6
Absorption correction: multi-scan (*SADABS*; Krause *et al.*, 2015)	*k* = −12→11
*T*_min_ = 0.84, *T*_max_ = 0.97	*l* = −16→17
5735 measured reflections	

### Refinement

**Table:**

Refinement on *F*^2^	Secondary atom site location: difference Fourier map
Least-squares matrix: full	Hydrogen site location: difference Fourier map
*R*[*F*^2^ > 2σ(*F*^2^)] = 0.041	All H-atom parameters refined
*wR*(*F*^2^) = 0.100	*w* = 1/[σ^2^(*F*_o_^2^) + (0.0403*P*)^2^ + 0.2108*P*] where *P* = (*F*_o_^2^ + 2*F*_c_^2^)/3
*S* = 1.07	(Δ/σ)_max_ < 0.001
2764 reflections	Δρ_max_ = 0.25 e Å^−^^3^
276 parameters	Δρ_min_ = −0.21 e Å^−^^3^
0 restraints	Extinction correction: *SHELXL2018* (Sheldrick, 2015*b*), Fc^*^=kFc[1+0.001xFc^2^λ^3^/sin(2θ)]^-1/4^
Primary atom site location: structure-invariant direct methods	Extinction coefficient: 0.0091 (10)

### Special details

**Table:**

Geometry. All esds (except the esd in the dihedral angle between two l.s. planes) are estimated using the full covariance matrix. The cell esds are taken into account individually in the estimation of esds in distances, angles and torsion angles; correlations between esds in cell parameters are only used when they are defined by crystal symmetry. An approximate (isotropic) treatment of cell esds is used for estimating esds involving l.s. planes.
Refinement. Refinement of F^2^ against ALL reflections. The weighted R-factor wR and goodness of fit S are based on F^2^, conventional R-factors R are based on F, with F set to zero for negative F^2^. The threshold expression of F^2^ > 2sigma(F^2^) is used only for calculating R-factors(gt) etc. and is not relevant to the choice of reflections for refinement. R-factors based on F^2^ are statistically about twice as large as those based on F, and R- factors based on ALL data will be even larger.

### Fractional atomic coordinates and isotropic or equivalent isotropic displacement parameters (Å^2^)

**Table:**

	*x*	*y*	*z*	*U*_iso_*/*U*_eq_	
O1	0.1373 (2)	0.25655 (12)	0.71106 (8)	0.0317 (3)	
N1	0.6669 (2)	0.43499 (13)	0.88772 (9)	0.0246 (3)	
N2	0.4435 (2)	0.41368 (12)	0.69511 (8)	0.0214 (3)	
N3	0.4218 (2)	0.10144 (13)	0.42094 (8)	0.0214 (3)	
N4	0.6468 (2)	0.17916 (13)	0.43816 (9)	0.0257 (3)	
N5	0.6307 (2)	0.29436 (13)	0.50153 (9)	0.0253 (3)	
C1	0.6701 (3)	0.49930 (15)	0.73511 (10)	0.0214 (3)	
C2	0.7938 (3)	0.57499 (16)	0.68289 (11)	0.0245 (3)	
H2	0.722 (3)	0.5669 (19)	0.6158 (13)	0.034 (5)*	
C3	1.0190 (3)	0.65725 (16)	0.72646 (12)	0.0282 (4)	
H3	1.102 (3)	0.7133 (19)	0.6888 (13)	0.032 (5)*	
C4	1.1277 (3)	0.66500 (17)	0.82210 (12)	0.0291 (4)	
H4	1.280 (4)	0.720 (2)	0.8511 (14)	0.039 (5)*	
C5	1.0094 (3)	0.59047 (16)	0.87417 (11)	0.0267 (3)	
H5	1.084 (3)	0.5905 (18)	0.9413 (13)	0.031 (5)*	
C6	0.7797 (3)	0.50745 (15)	0.83176 (10)	0.0227 (3)	
C7	0.4566 (3)	0.35724 (15)	0.84935 (10)	0.0234 (3)	
C8	0.3301 (3)	0.33717 (15)	0.74734 (10)	0.0231 (3)	
C9	0.3266 (3)	0.28202 (18)	0.90846 (12)	0.0302 (4)	
H9A	0.422 (4)	0.301 (2)	0.9742 (15)	0.046 (6)*	
H9B	0.310 (4)	0.182 (2)	0.8803 (15)	0.051 (6)*	
H9C	0.153 (4)	0.304 (2)	0.9088 (14)	0.048 (6)*	
C10	0.3087 (3)	0.40222 (16)	0.59533 (10)	0.0232 (3)	
H10A	0.338 (3)	0.4926 (18)	0.5805 (12)	0.025 (4)*	
H10B	0.125 (3)	0.3822 (17)	0.5929 (12)	0.028 (4)*	
C11	0.3937 (3)	0.28917 (15)	0.52445 (10)	0.0207 (3)	
C12	0.2596 (3)	0.16668 (16)	0.47318 (10)	0.0222 (3)	
H12	0.088 (4)	0.1281 (19)	0.4678 (13)	0.033 (5)*	
C13	0.3800 (3)	−0.03351 (16)	0.35173 (11)	0.0265 (3)	
H13A	0.551 (3)	−0.0671 (18)	0.3519 (12)	0.030 (4)*	
H13B	0.280 (4)	−0.096 (2)	0.3780 (13)	0.036 (5)*	
C14	0.2469 (3)	−0.02638 (17)	0.25207 (11)	0.0262 (3)	
H14A	0.210 (3)	−0.123 (2)	0.2122 (13)	0.034 (5)*	
H14B	0.078 (3)	0.0099 (18)	0.2559 (12)	0.031 (5)*	
C15	0.3969 (3)	0.05954 (18)	0.20269 (11)	0.0293 (4)	
H15A	0.570 (4)	0.0313 (19)	0.2062 (13)	0.037 (5)*	
H15B	0.412 (3)	0.160 (2)	0.2368 (13)	0.035 (5)*	
C16	0.2667 (4)	0.0438 (2)	0.09843 (13)	0.0420 (5)	
H16A	0.248 (4)	−0.057 (2)	0.0616 (15)	0.047 (6)*	
H16B	0.360 (5)	0.095 (3)	0.0645 (17)	0.067 (7)*	
H16C	0.092 (5)	0.071 (3)	0.0943 (17)	0.068 (7)*	

### Atomic displacement parameters (Å^2^)

**Table:**

	*U*^11^	*U*^22^	*U*^33^	*U*^12^	*U*^13^	*U*^23^
O1	0.0292 (6)	0.0338 (7)	0.0292 (6)	−0.0076 (5)	0.0029 (5)	0.0062 (5)
N1	0.0267 (7)	0.0249 (7)	0.0226 (6)	0.0033 (5)	0.0053 (5)	0.0059 (5)
N2	0.0216 (6)	0.0233 (7)	0.0191 (6)	0.0024 (5)	0.0035 (5)	0.0048 (5)
N3	0.0177 (6)	0.0237 (7)	0.0215 (6)	0.0004 (5)	0.0013 (4)	0.0051 (5)
N4	0.0193 (6)	0.0290 (7)	0.0255 (6)	−0.0012 (5)	0.0031 (5)	0.0017 (5)
N5	0.0212 (6)	0.0286 (7)	0.0246 (6)	−0.0006 (5)	0.0054 (5)	0.0030 (5)
C1	0.0205 (7)	0.0208 (7)	0.0227 (7)	0.0034 (5)	0.0055 (5)	0.0032 (6)
C2	0.0273 (8)	0.0238 (8)	0.0240 (8)	0.0050 (6)	0.0063 (6)	0.0071 (6)
C3	0.0287 (8)	0.0261 (8)	0.0326 (8)	0.0015 (6)	0.0113 (6)	0.0090 (7)
C4	0.0234 (8)	0.0284 (8)	0.0330 (8)	−0.0026 (6)	0.0049 (6)	0.0038 (7)
C5	0.0252 (8)	0.0291 (8)	0.0234 (8)	0.0019 (6)	0.0026 (6)	0.0029 (6)
C6	0.0232 (7)	0.0224 (8)	0.0229 (7)	0.0030 (6)	0.0062 (6)	0.0046 (6)
C7	0.0265 (8)	0.0209 (8)	0.0236 (7)	0.0042 (6)	0.0067 (6)	0.0054 (6)
C8	0.0235 (7)	0.0227 (8)	0.0239 (7)	0.0033 (6)	0.0068 (6)	0.0052 (6)
C9	0.0348 (9)	0.0297 (9)	0.0268 (8)	−0.0017 (7)	0.0071 (7)	0.0084 (7)
C10	0.0219 (7)	0.0264 (8)	0.0208 (7)	0.0037 (6)	0.0021 (6)	0.0057 (6)
C11	0.0182 (7)	0.0260 (8)	0.0194 (7)	0.0025 (5)	0.0027 (5)	0.0091 (6)
C12	0.0164 (7)	0.0282 (8)	0.0226 (7)	0.0004 (6)	0.0037 (5)	0.0077 (6)
C13	0.0294 (8)	0.0205 (8)	0.0269 (8)	0.0011 (6)	0.0024 (6)	0.0029 (6)
C14	0.0235 (8)	0.0261 (8)	0.0251 (8)	−0.0013 (6)	0.0007 (6)	0.0021 (6)
C15	0.0307 (9)	0.0296 (9)	0.0263 (8)	−0.0005 (7)	0.0050 (6)	0.0049 (7)
C16	0.0528 (12)	0.0436 (12)	0.0286 (9)	−0.0001 (9)	0.0044 (8)	0.0101 (8)

### Geometric parameters (Å, º)

**Table:**

O1—C8	1.2292 (18)	C7—C9	1.492 (2)
N1—C7	1.2891 (19)	C9—H9A	0.97 (2)
N1—C6	1.3917 (19)	C9—H9B	0.98 (2)
N2—C8	1.3780 (19)	C9—H9C	0.96 (2)
N2—C1	1.3951 (18)	C10—C11	1.495 (2)
N2—C10	1.4787 (17)	C10—H10A	0.986 (18)
N3—N4	1.3451 (17)	C10—H10B	0.982 (18)
N3—C12	1.3471 (18)	C11—C12	1.368 (2)
N3—C13	1.4690 (19)	C12—H12	0.954 (19)
N4—N5	1.3196 (18)	C13—C14	1.517 (2)
N5—C11	1.3617 (18)	C13—H13A	0.986 (18)
C1—C2	1.401 (2)	C13—H13B	0.98 (2)
C1—C6	1.407 (2)	C14—C15	1.515 (2)
C2—C3	1.382 (2)	C14—H14A	0.996 (19)
C2—H2	0.966 (18)	C14—H14B	0.992 (18)
C3—C4	1.393 (2)	C15—C16	1.522 (2)
C3—H3	1.003 (19)	C15—H15A	0.977 (19)
C4—C5	1.377 (2)	C15—H15B	1.005 (19)
C4—H4	0.93 (2)	C16—H16A	1.02 (2)
C5—C6	1.401 (2)	C16—H16B	0.96 (3)
C5—H5	0.988 (18)	C16—H16C	0.98 (3)
C7—C8	1.482 (2)		
			
O1···C11	3.3134 (19)	C4···C8^iv^	3.502 (2)
O1···C13^i^	3.306 (2)	C5···C8^iv^	3.491 (2)
O1···C14^i^	3.193 (2)	C5···C7^iv^	3.429 (2)
O1···H10B	2.341 (17)	C11···C13^ii^	3.589 (2)
O1···H9B	2.75 (2)	C12···C13^ii^	3.470 (2)
O1···H9C	2.79 (2)	C16···C16^viii^	3.577 (3)
O1···H13A^ii^	2.696 (17)	C2···H15B^vii^	2.991 (19)
O1···H13B^i^	2.62 (2)	C2···H10A	2.600 (17)
O1···H14A^i^	2.752 (18)	C2···H10B^iv^	2.928 (17)
N1···N2	2.8013 (17)	C3···H15B^vii^	3.055 (18)
N2···C3^iii^	3.421 (2)	C4···H16C^vii^	3.03 (3)
N2···N5	3.1514 (17)	C7···H14A^ii^	3.081 (19)
N4···C15	3.364 (2)	C8···H13A^ii^	2.918 (18)
N4···C12^iv^	3.228 (2)	C10···H2	2.608 (18)
N5···C2	3.343 (2)	C11···H2^vii^	2.755 (19)
N1···H5^v^	2.680 (18)	C11···H13B^ii^	3.08 (2)
N3···H15B	2.860 (19)	C11···H2	3.086 (19)
N4···H12^iv^	2.42 (2)	C12···H13A^ii^	2.973 (17)
N4···H13B^ii^	2.944 (19)	C15···H4^vi^	3.04 (2)
N4···H15B	2.936 (18)	C15···H9B^ii^	3.08 (2)
N4···H3^vi^	2.821 (18)	C16···H16C^viii^	3.04 (2)
N5···H2	2.819 (19)	H2···H10A	2.07 (2)
N5···H10B^iv^	2.733 (17)	H4···H16B^vi^	2.52 (3)
N5···H2^vii^	2.932 (18)	H9B···H15A^ii^	2.40 (3)
N5···H10A^vii^	2.676 (18)	H13A···H15A	2.54 (3)
N5···H13B^ii^	2.93 (2)	H14A···H16A	2.46 (3)
C2···C10^iv^	3.586 (2)	H14B···H16C	2.57 (3)
C2···C11	3.559 (2)	H15A···H13A	2.54 (3)
C2···C11^vii^	3.589 (2)	H15A···H9B^ii^	2.40 (3)
C3···C10^iv^	3.431 (2)		
			
C7—N1—C6	118.81 (13)	H9B—C9—H9C	104.4 (17)
C8—N2—C1	121.65 (12)	N2—C10—C11	112.53 (12)
C8—N2—C10	116.29 (12)	N2—C10—H10A	107.5 (10)
C1—N2—C10	122.04 (12)	C11—C10—H10A	111.5 (10)
N4—N3—C12	110.55 (12)	N2—C10—H10B	107.9 (10)
N4—N3—C13	120.31 (12)	C11—C10—H10B	108.4 (10)
C12—N3—C13	129.14 (13)	H10A—C10—H10B	108.9 (14)
N5—N4—N3	107.55 (11)	N5—C11—C12	108.52 (13)
N4—N5—C11	108.38 (12)	N5—C11—C10	123.12 (13)
N2—C1—C2	122.89 (13)	C12—C11—C10	128.37 (13)
N2—C1—C6	118.01 (13)	N3—C12—C11	105.01 (13)
C2—C1—C6	119.10 (14)	N3—C12—H12	121.9 (11)
C3—C2—C1	120.11 (14)	C11—C12—H12	133.1 (11)
C3—C2—H2	120.6 (11)	N3—C13—C14	112.79 (13)
C1—C2—H2	119.3 (11)	N3—C13—H13A	105.8 (10)
C2—C3—C4	120.75 (15)	C14—C13—H13A	112.3 (10)
C2—C3—H3	119.2 (10)	N3—C13—H13B	107.0 (11)
C4—C3—H3	120.0 (10)	C14—C13—H13B	111.2 (11)
C5—C4—C3	119.81 (15)	H13A—C13—H13B	107.3 (15)
C5—C4—H4	119.6 (12)	C15—C14—C13	114.99 (13)
C3—C4—H4	120.6 (12)	C15—C14—H14A	109.5 (10)
C4—C5—C6	120.42 (14)	C13—C14—H14A	107.1 (10)
C4—C5—H5	122.2 (11)	C15—C14—H14B	109.1 (10)
C6—C5—H5	117.3 (11)	C13—C14—H14B	109.3 (10)
N1—C6—C5	118.15 (13)	H14A—C14—H14B	106.5 (14)
N1—C6—C1	122.05 (13)	C14—C15—C16	111.11 (14)
C5—C6—C1	119.80 (14)	C14—C15—H15A	109.0 (11)
N1—C7—C8	123.58 (14)	C16—C15—H15A	110.5 (11)
N1—C7—C9	120.01 (13)	C14—C15—H15B	110.4 (10)
C8—C7—C9	116.41 (13)	C16—C15—H15B	108.5 (10)
O1—C8—N2	121.86 (13)	H15A—C15—H15B	107.3 (15)
O1—C8—C7	122.32 (14)	C15—C16—H16A	110.7 (12)
N2—C8—C7	115.82 (13)	C15—C16—H16B	113.1 (14)
C7—C9—H9A	111.1 (12)	H16A—C16—H16B	106.6 (17)
C7—C9—H9B	110.3 (12)	C15—C16—H16C	111.0 (14)
H9A—C9—H9B	109.1 (17)	H16A—C16—H16C	105.7 (18)
C7—C9—H9C	112.1 (12)	H16B—C16—H16C	109 (2)
H9A—C9—H9C	109.6 (16)		
			
C12—N3—N4—N5	0.11 (16)	C1—N2—C8—O1	−176.40 (13)
C13—N3—N4—N5	179.17 (12)	C10—N2—C8—O1	4.9 (2)
N3—N4—N5—C11	0.03 (15)	C1—N2—C8—C7	2.99 (19)
C8—N2—C1—C2	178.01 (13)	C10—N2—C8—C7	−175.71 (12)
C10—N2—C1—C2	−3.4 (2)	N1—C7—C8—O1	175.85 (14)
C8—N2—C1—C6	−1.0 (2)	C9—C7—C8—O1	−4.4 (2)
C10—N2—C1—C6	177.67 (13)	N1—C7—C8—N2	−3.5 (2)
N2—C1—C2—C3	−179.52 (13)	C9—C7—C8—N2	176.18 (13)
C6—C1—C2—C3	−0.6 (2)	C8—N2—C10—C11	−90.45 (15)
C1—C2—C3—C4	0.7 (2)	C1—N2—C10—C11	90.85 (16)
C2—C3—C4—C5	−0.3 (2)	N4—N5—C11—C12	−0.15 (16)
C3—C4—C5—C6	−0.3 (2)	N4—N5—C11—C10	179.35 (13)
C7—N1—C6—C5	−179.41 (14)	N2—C10—C11—N5	−69.14 (18)
C7—N1—C6—C1	0.5 (2)	N2—C10—C11—C12	110.25 (16)
C4—C5—C6—N1	−179.55 (14)	N4—N3—C12—C11	−0.20 (15)
C4—C5—C6—C1	0.5 (2)	C13—N3—C12—C11	−179.15 (13)
N2—C1—C6—N1	−1.0 (2)	N5—C11—C12—N3	0.22 (16)
C2—C1—C6—N1	180.00 (13)	C10—C11—C12—N3	−179.25 (13)
N2—C1—C6—C5	178.96 (13)	N4—N3—C13—C14	−95.26 (16)
C2—C1—C6—C5	0.0 (2)	C12—N3—C13—C14	83.59 (19)
C6—N1—C7—C8	1.8 (2)	N3—C13—C14—C15	63.83 (18)
C6—N1—C7—C9	−177.94 (14)	C13—C14—C15—C16	171.75 (15)

Symmetry codes: (i) −*x*, −*y*, −*z*+1; (ii) −*x*+1, −*y*, −*z*+1; (iii) *x*−1, *y*, *z*; (iv) *x*+1, *y*, *z*; (v) −*x*+2, −*y*+1, −*z*+2; (vi) −*x*+2, −*y*+1, −*z*+1; (vii) −*x*+1, −*y*+1, −*z*+1; (viii) −*x*, −*y*, −*z*.

### Hydrogen-bond geometry (Å, º)

Cg1 is the centroid of the N3–N5/C11/C12 ring.

**Table:**

*D*—H···*A*	*D*—H	H···*A*	*D*···*A*	*D*—H···*A*
C12—H12···N4^iii^	0.954 (19)	2.419 (19)	3.2282 (19)	142.4 (15)
C2—H2···*Cg*1^vii^	0.966 (18)	2.986 (19)	3.642 (1)	126.3 (14)

Symmetry codes: (iii) *x*−1, *y*, *z*; (vii) −*x*+1, −*y*+1, −*z*+1.
